# Percutaneous Zero-fluoroscopy Atrial Septal Defect Closure *Versus* Fluoroscopy-guided Method: A Systematic Review and Meta-analysis

**DOI:** 10.2174/011573403X337854250227052833

**Published:** 2025-03-06

**Authors:** Brian Mendel, Kelvin Kohar, Richie Jonathan Djiu, Defin Allevia Yumnanisha, Justin Winarta, Gusti Ngurah Prana Jagannatha, Theresia Feline Husen, Sisca Natalia Siagian, Radityo Prakoso

**Affiliations:** 1 Department of Cardiology and Vascular Medicine, National Cardiovascular Center Harapan Kita, Universitas Indonesia, Jakarta, Indonesia;; 2 Faculty of Medicine, Universitas Indonesia, Jakarta, Indonesia;; 3 Faculty of Medicine, Prof. Dr. I.G.N.G Ngoerah General Hospital, Udayana University, Denpasar, Bali, Indonesia;; 4 Division of Pediatric Cardiology and Congenital Heart Disease, Department of Cardiology and Vascular Medicine, National Cardiovascular Center Harapan Kita, Universitas Indonesia, Jakarta, Indonesia

**Keywords:** Atrial septal defect, echocardiography, percutaneous, success rate, zero fluoroscopy, congenital heart disease

## Abstract

**Introduction:**

Percutaneous atrial septal defects (ASD) closure with fluoroscopy guidance is the standard procedure. However, fluoroscopy poses stochastic and deterministic risks for small infants and children. Zero fluoroscopy ASD closure is an alternative, yet its feasibility and safety compared to fluoroscopy remain unclear. Therefore, this study compares outcomes using standardized fluoroscopy and zero fluoroscopy methods for transcatheter ASD closure.

**Methods:**

Four databases (PubMed, ProQuest, Google Scholar, Wiley) were used to search literature published before July 2023. The main results were the success rate and the complications. Outcomes were processed using the DerSimonian-Laird random-effects model of proportional meta-analysis to determine the overall proportion.

**Results:**

A total of 68 cohort studies (8,989 patients) were included in this meta-analysis. Overall, percutaneous ASD closure was successfully performed in 97% of patients (95%CI: 96-98%) based on 59 studies (8,989 patients), of which fluoroscopy accounted for 97% (95%CI: 96-98%) based on 51 studies (7,760 patients) and non-fluoroscopy for 98% (95%CI: 96-100%)] based on 8 studies (1,229 patients). Device embolization, AV block, and other arrhythmias did not differ significantly between the two groups. However, the percentage difference in residual leaks between the two groups was quite vast, with 5% in the non-fluoroscopy group and 12% in the fluoroscopy group.

**Conclusion:**

Percutaneous ASD closure with zero fluoroscopy is safe and effective, as evidenced by the high success rate, and is non-inferior to the standardized fluoroscopy method.

## 1 INTRODUCTION

The secundum form of atrial septal defect (ASD) accounts for 6-10% of congenital heart disease (CHD) [1]. Left to right shunt in ASD may result in considerable clinical disability if left untreated, particularly as children become adults [2]. Since the initial report of transcatheter ASD repair four decades ago, advances in implant techniques, materials, and device design have proven that transcatheter closure is superior to conventional open-heart surgery [3, 4]. Percutaneous ASD closure also serves as a less invasive approach for individuals with secundum defects and those heavily burdened by concomitant illnesses, for whom surgery entails a higher risk.

However, fluoroscopic-guided percutaneous closure can be problematic because patients must be exposed to medical radiation numerous times, which is associated with a spectrum of malignancies, particularly in pregnant women and children [5-8]. Transesophageal echocardiography (TEE) was first applied as an adjunct to X-ray imaging for intraoperative cardiac surgery evaluation and catheter guidance. TEE has been shown to be practical in recent years for directing transcatheter ASD closure in children and expectant mothers [5-8]. Over the years, periprocedural 2D and 3D transthoracic echocardiography TEE has become more accurate in terms of imaging characteristics [9]. Moreover, with time, a deeper comprehension of the hemodynamic significance of atrial shunts ascertained by imaging methods has emerged.

Nevertheless, the results of ASD closure with a percutaneous device only guided by echocardiography or zero fluoroscopy are still not well understood [8]. There are still uncertainties related to success rates and complications between both guiding methods. Therefore, in this meta-analysis, we compared the standard fluoroscopy-guided percutaneous closure to the procedural success and complications of zero fluoroscopy percutaneous closure.

## 2 METHODS

This systematic review and meta-analysis were conducted in accordance with the Cochrane Handbook for Systematic Reviews of Interventions and aligned with the Preferred Reporting Items for Systematic Reviews and Meta-Analyses (PRISMA) guidelines [10]. The study follows the principles outlined in the Declaration of Helsinki, a standard consistently upheld in all studies cited within this paper.

### 2.1 Search Strategy

A literature search for relevant journals published before July 2023 was conducted on PubMed, ProQuest, Google Scholar, and Wiley. The search included the keywords (ASD OR atrial septal defect) AND (percutaneous closure) AND (Fluoroscopy OR Echocardiography OR TEE OR transesophageal echocardiogram OR TTE OR transthoracic echocardiogram). Relevant papers from these sources were examined.

### 2.2 Study Eligibility Criteria and Screening Process

The study eligibility criteria, following the PICO framework, included (1) Type of study: Cohort; (2) Study population: ASD patients; (3) Intervention: percutaneous ASD closure; (4) Outcomes: success rate and overall complication. Procedural success was defined as a device successfully delivered and placed in the atrial septal defect. We did not specify an age limitation in the patient inclusion criteria, as the procedure can be performed regardless of age. Concurrently, the exclusion criteria were defined as follows: (1) incomplete studies at the time of retrieval; (2) full-text publications that cannot be recovered; and (3) research conducted in languages other than English. The entire search and screening process was done using Google Sheets (Google LLC, Mountain View, CA). Six reviewers (BM, KK, RJD, DAY, JW, and TFH) screened study titles and abstracts separately, following the eligibility requirements. Any disagreements were discussed and resolved.

### 2.3 Data Extraction

Six authors (BM, KK, RJD, DAY, JW, and TFH) separately retrieved the relevant data in Google Sheets (Google LLC). The data retrieved included the following: (1) author of the study; (2) characteristics of the study, including study design, study location, and study interval; (3) population characteristics, including the number of patients, age, weight, ASD size, and rim, and comorbidities; (4) intervention characteristics, including type and size of the devices used, methods of guiding closure, and follow-up period; and (5) interest outcomes, including technique success, post-procedure complications, and mortality (Supporting information).

### 2.4 Quantitative Data Analysis

Each table and figure summarizes the data synthesis. The technique success and post-procedure complications were outcome-related procedure variables that were characterized using fundamental relative frequency and proportions, which are calculated as the number of cases divided by the population size. The DerSimonian-Laird approach and a random-effects model were used for a proportional meta-analysis. I^2^ statistics, ranging from 0% to 100%, were used to evaluate statistical heterogeneity. We define low heterogeneity as I^2^ <25%, moderate heterogeneity as 25-50%, and severe heterogeneity as I^2^ >50%. An objective Egger's test and a funnel plot were used to evaluate publication bias. RStudio version 2022.07.2+576 was utilized for all analyses [11]. We also performed subgroup analyses based on two factors: age (children and adults) and procedure complications. Children were defined as individuals under 18 years old, and adults as those 18 years or older. Procedure complications were further subgrouped into device embolization, arrhythmia, AV block, and residual leak.

### 2.5 Risk of Bias Assessment

Strengthening the Reporting of Observational Studies in Epidemiology (STROBE) was used by six authors (BM, KK, RJD, DAY, JW, and TFH) to evaluate each independently included study's likelihood of bias since the included studies are observational non-randomized studies. STROBE traffic light plots, comprising all of its domains, were the result. The colors green, yellow, and red denoted low, some concerns, and high risk of bias [12].

## 3 RESULTS

### 3.1 Study Selections

We identified a total of 55,110 studies upon initial search. Initial screening of the title and abstract revealed 115 studies eligible for full-text review. Among these, 46 studies were excluded for various reasons: six due to inaccessibility and 43 due to insufficient data. Finally, 68 studies with a total of 8,989 patients were included in this systematic review and meta-analysis [13-80]. The selection process is depicted in Fig. (**1**). Each of the studies was then critically appraised using the STROBE statement.

### 3.2 Study Characteristics

The included studies came from various locations over different periods, ranging from 2001 to 2020. Sixty-eight studies were conducted in 23 countries, most of them in the United States (American region), United Kingdom (European region), and China (Asian region). Most included studies were cohorts (Fifty retrospective and sixteen prospective cohorts), and the other two were clinical trials [13-82].

A total of 8,989 patients (children and adults) underwent percutaneous ASD closure, with 7,760 patients having fluoroscopy guidance and 1,229 having non-fluoroscopy guidance. The devices used in the included studies were Amplatzer septal occluder, followed by other devices, such as BioSTAR implant, HELEX septal occluder, GORE Cardioform ASD, *etc*. Percutaneous defect closure was performed using fluoroscopy and a non-fluoroscopy approach (TTE or TEE). Mean follow-up time varied from six months to three years (Supporting information).

### 3.3 Synthesis of Results

Sixty studies utilized fluoroscopy, six studies utilized zero fluoroscopy, and two studies reported both separately. Overall, percutaneous ASD closure saw a success rate of 97% (95%CI: 96-98%) based on 59 studies (8,989 patients) depicted in Fig. (**2**), whereas the success rate was similar between fluoroscopy and non-fluoroscopy approaches, which was 97% (95%CI: 96-98%) based on 51 studies (7,760 patients) and 98% (95%CI: 96-100%)] based on 8 studies (1,229 patients). Further, the test between subgroups revealed no significant difference (*p* = 0.59). However, high heterogeneity was found in both results (I^2^ = 79% and 77%).

**Fig. (3):**
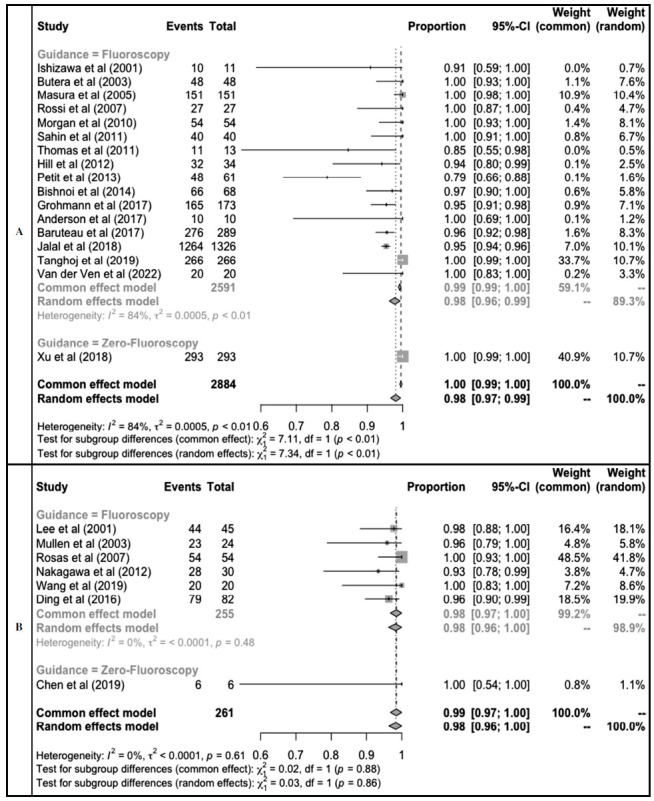
Proportional meta-analysis of percutaneous ASD closure success rate in children **(A)** and adults **(B)**.

Subgroup analysis of success rate was also performed in a population of children and adults. The overall success rate of percutaneous closure in children was 98% (95%CI: 97-99%). Fluoroscopy guidance in children was performed in 16 studies (2,591 participants) with a success rate of 98% (95%CI: 96-99%) participants. Meanwhile, only one study performed a zero-fluoroscopy procedure in children with a success rate of 100%. In adults, the overall success rate was 98% (95%CI: 96-100%) from seven studies. Six studies performed ASD closure using fluoroscopy guidance with a success rate of 98% (95%CI: 96-100%), and only 1 study performed zero-fluoroscopy with a success rate of 100% (Fig. **3**).

**Fig. (4):**
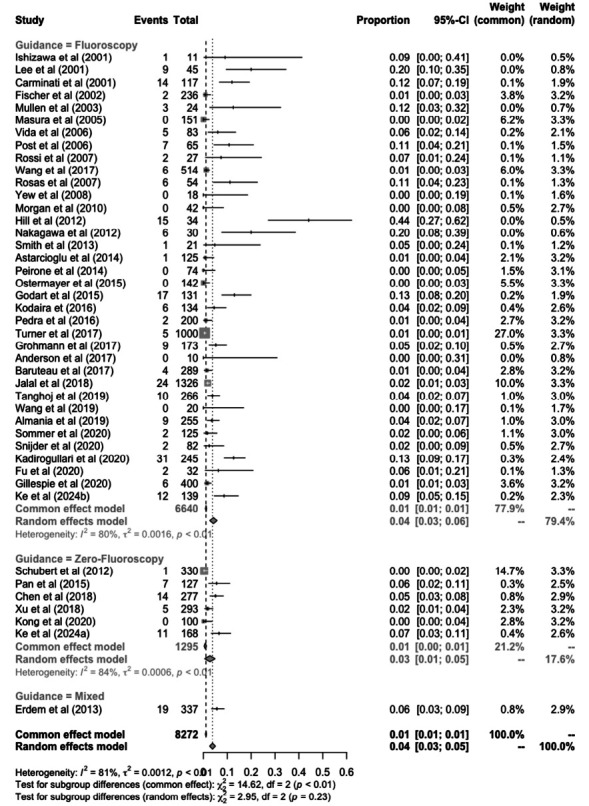
Proportional meta-analysis of procedure complications.

**Fig. (5):**
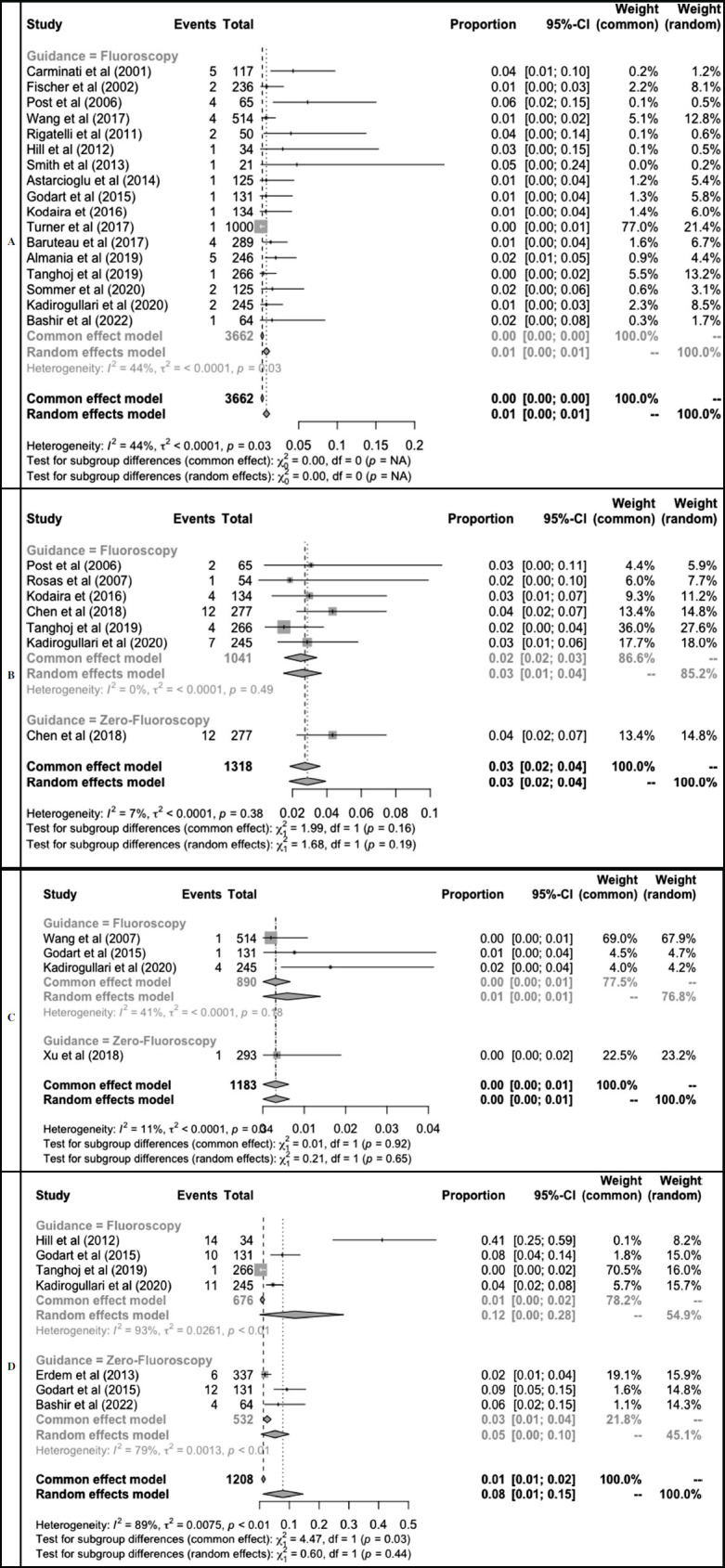
Proportional meta-analysis of complications: device embolization **(A)**, arrhythmia **(B)**, AV block **(C)**, and residual leak **(D).** Device embolization was reported in 1% of fluoroscopy patients; however, no studies reported this outcome in the non-fluoroscopy group. Patients in the non-fluoroscopy group presented with a non-significantly lower rate of residual leak [5% (95%CI: 0-15%) and 12% (95%CI: 1-28%), *p* = 0.4]. A total of 4% of patients in the non-fluoroscopy group presented with arrhythmia. The arrhythmia rate was non-significantly higher compared to the fluoroscopy group [3% (95%CI: 1-4%), test of subgroup differences *p* = 0.19].

Despite the success of both interventions, complications were discovered in some cases (Figs. **4** and **5**). The complications rate was non-significantly lower (*p* = 0.23) in the non-fluoroscopy group [3% (95%CI: 1-5%) compared to the fluoroscopy group [4% (95%CI: 3-6%)] (Fig. **4** and Table **1**). Device embolization was reported in 1% of fluoroscopy patients, however, no studies reported this outcome in the non-fluoroscopy group. Patients in the non-fluoroscopy group presented with a non-significantly lower rate of residual leak [5% (95%CI: 0-15%) and 12% (95%CI: 1-28%), *p* = 0.^4^]. A total of 4% of patients in the non-fluoroscopy group presented with arrhythmia, as reported by Chen *et al*. (2018). The arrhythmia rate was non-significantly higher compared to the fluoroscopy group [3% (95%CI: 1-4%), test of subgroup differences *p* = 0.^19^]. Xu *et al.* (2018) reported one patient in a non-fluoroscopy group having a post-procedural AV block (Fig. **5**).

### 3.4 Publication Bias

The meta-analysis yielded a *p*-value<0.001, indicating a low likelihood of publication bias. The details are shown in Fig. (**6**).

## 4 DISCUSSION

Medical X-rays, particularly cardiac catheterizations, are commonly used to treat CHD. The impact of radiation exposure at young ages and the risk of cancer in CHD patients is still being debated, particularly in cases where many procedures utilizing ionizing radiation are performed [83, 84]. The tissues of growing children are more vulnerable to the harmful effects of radiation than adult tissue because of their higher mitotic activity. Furthermore, children are more vulnerable to radiation-induced sickness than adults since they have a longer life expectancy and more time for the consequences to manifest [85, 86]. Borghini *et al*. (2023) found a strong link between pediatric radiation exposure from cardiac procedures and changes in early markers of genetic instability and carcinogenesis, as well as dysregulation of miR-155, a well-known carcinogenic miRNA [87].

For high radiation doses, cancer risk has been found to follow a “linear no-threshold” model, which asserts that cancer incidence increases linearly with dosage with no lower threshold [88]. This linear no-threshold model serves as the cornerstone of radiation safety for normal low-dose exposures to patients and workers. Radiation exposure poses significant professional health concerns to catheter lab operators and paramedical workers [89]. The typical lifetime work-related exposure is in the range of 50-200 mSv, equal to a whole-body dosage equivalent of 2500-10,000 chest X-rays (CXR) and an attributable excess cancer risk of 1 in 100 [89, 90]. Traditional interventional procedures were performed using fluoroscopy, and radiation exposure can directly disrupt biological macromolecules in the body, as well as the hematological, endocrine, reproductive, and other systems [91]. Furthermore, exposure to X radiation reduces the body's ability to neutralize oxygen free radicals and suppresses its antioxidant system, causing indirect injury. According to the study by Roguin *et al*. in 2013, 44 interventional cardiologists had brain and neck cancer on their left side, which is more frequently exposed to radiation than their right [92].

The American Society of Echocardiography (ASE) recently published a recommendation paper on the subject, which can be used for transcatheter structural heart disease intervention (TSHI), including the role of echocardiography in ASD closure [5]. Traditional echocardiogram competencies are distinguished from those relevant to interventional echocardiography since imaging is conducted and analyzed in real-time, necessitating efficient communication with other members of the TSHI team. Our meta-analysis found that zero fluoroscopy-only ASD closure yields comparable procedural success and complication rates to the fluoroscopy-guided method, independent of device types. This discovery is noteworthy, especially in view of concerns about radiation exposure and the risks connected with fluoroscopy. Additionally, our findings confirm that the non-fluoroscopic technique is not inferior to the fluoroscopic group. Furthermore, using a non-fluoroscopy approach reduces the possibility of radiation exposure, which provides significant benefits to both patients and operators.

Preprocedural TTE provides information on the type of defect, hemodynamic consequences from shunting, and other associated congenital heart problems [93, 94]. TEE, on the other hand, is vital in the detailed examination of the defect since it avoids bone and lung shadowing and, due to its proximity to the LA, allows for excellent pictures of atrial structures and the interatrial septum (IAS). TEE is required during ASD sizing and device deployment to ensure that the device disc and IAS are parallel [5]. Intracardiac echocardiography (ICE) also offers an additional method of intraprocedural imaging during ASD closure [93]. Anatomically clear real-time echocardiography monitoring allows for better observation and control of the occlusion device release process [80]. Even if the ASD is enormous size-wise, and the rim around the aortic valve or inferior vena cava is short or nonexistent, the procedure can still be carried out [95, 96].

**Fig. (7):**
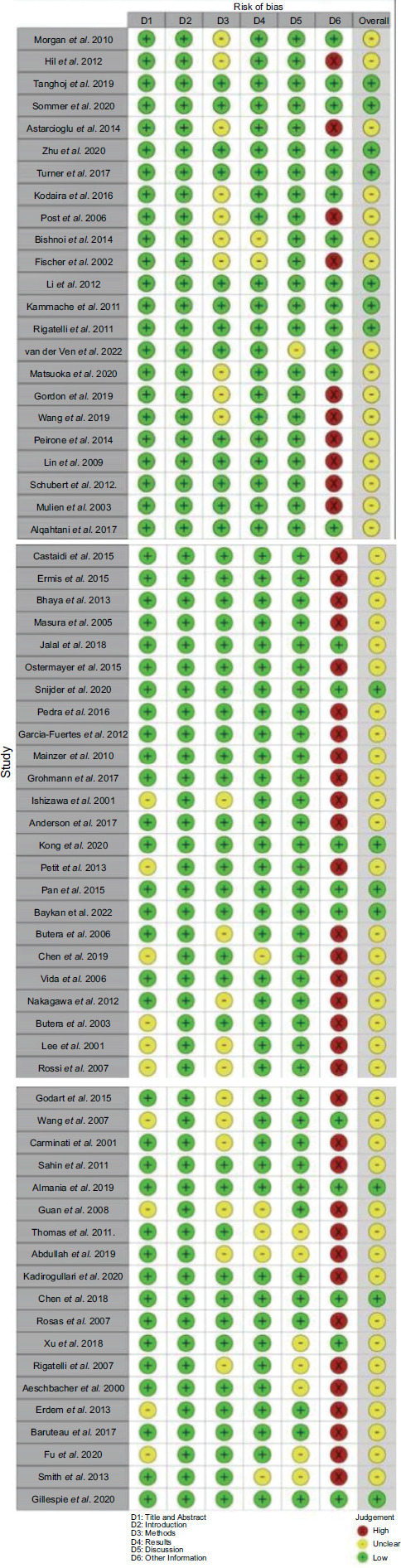
Critical appraisal of included studies.

The size of the ASD is crucial to the success of the procedure. In ASDs with a diameter of less than 25 mm, Zhu *et al*. (2020) demonstrated that percutaneous TEE-guided closure has a 100% success rate (Fig. **7**) [80]. The rim of the ASD, particularly one close to the vena cava, is crucial for straightforward TEE-guided percutaneous closure. The inferior vena cava must be at least 5 mm wide. Success rates are improved with 3 mm or thicker aortic valve rims [97]. Another critical element in successfully closing the ASD is the size of the occluder. The occluder size should be the greatest diameter of the defect plus 6 mm for a typical TEE-guided percutaneous ASD closure in order to maximize success rates. The size of the occluder should be the longest diameter of the defect plus 4 mm when the ASD is less than 25 mm or 4-6 mm when the longest diameter of the ASD is greater than 25 mm [80, 93].

Tracking the guidewire, catheter, and delivery sheath in the two-dimensional echocardiographic view plane, which is related to safety and procedure time, is the biggest challenge of zero fluoroscopy in comparison to fluoroscopic guidance. The position of the catheter is easily determined using conventional intervention procedures because fluoroscopy can identify it through projection. Accuracy would be significantly improved by having a thorough grasp of the anatomy and physiology of the heart and by using the proper guidewire, catheter, delivery sheath, and ultrasound probe placement [8]. The size and nearby structures of the ASD should be taken into consideration when developing pre-procedural selection criteria for zero fluoroscopy transcatheter ASD closure [98].

## CONCLUSION

Percutaneous zero fluoroscopy ASD closure techniques have shown comparable success rates and procedural outcomes to fluoroscopy-guided procedures. Future randomized controlled trials comparing both guidance modalities are needed to better understand the function of interventional echocardiography in ASD closure, in accordance with the American Society of Echocardiography (ASE) recommendation.

## LIMITATION

Since percutaneous non-fluoroscopy ASD closure is a relatively new discovery, there was a sample size imbalance in this meta-analysis, which may limit the ability to detect significant differences between the two groups. Furthermore, all studies were observational, making them susceptible to confounding and selection bias. Individual institutions are also likely to have diverse strategies for intervention and reporting.

## Figures and Tables

**Fig. (1):**
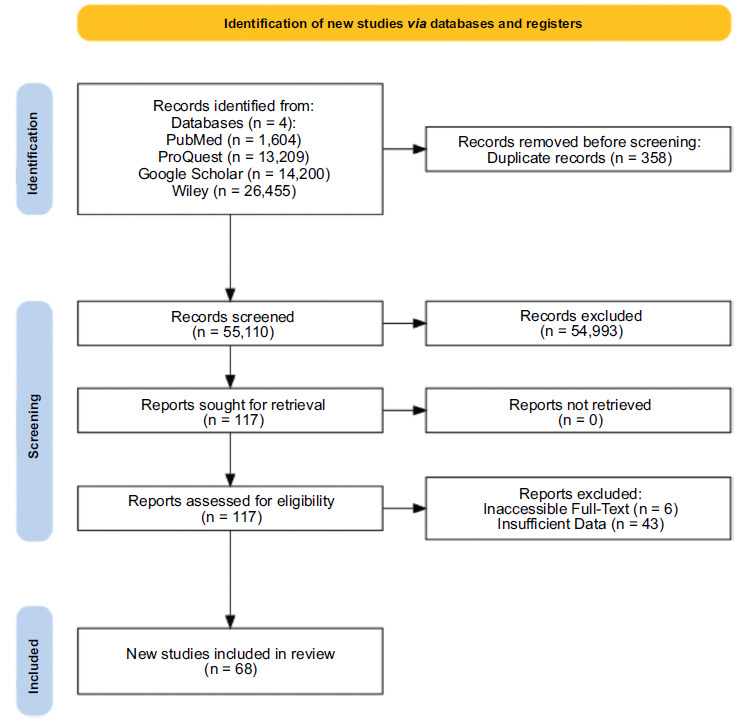
PRISMA flowchart.

**Fig. (2):**
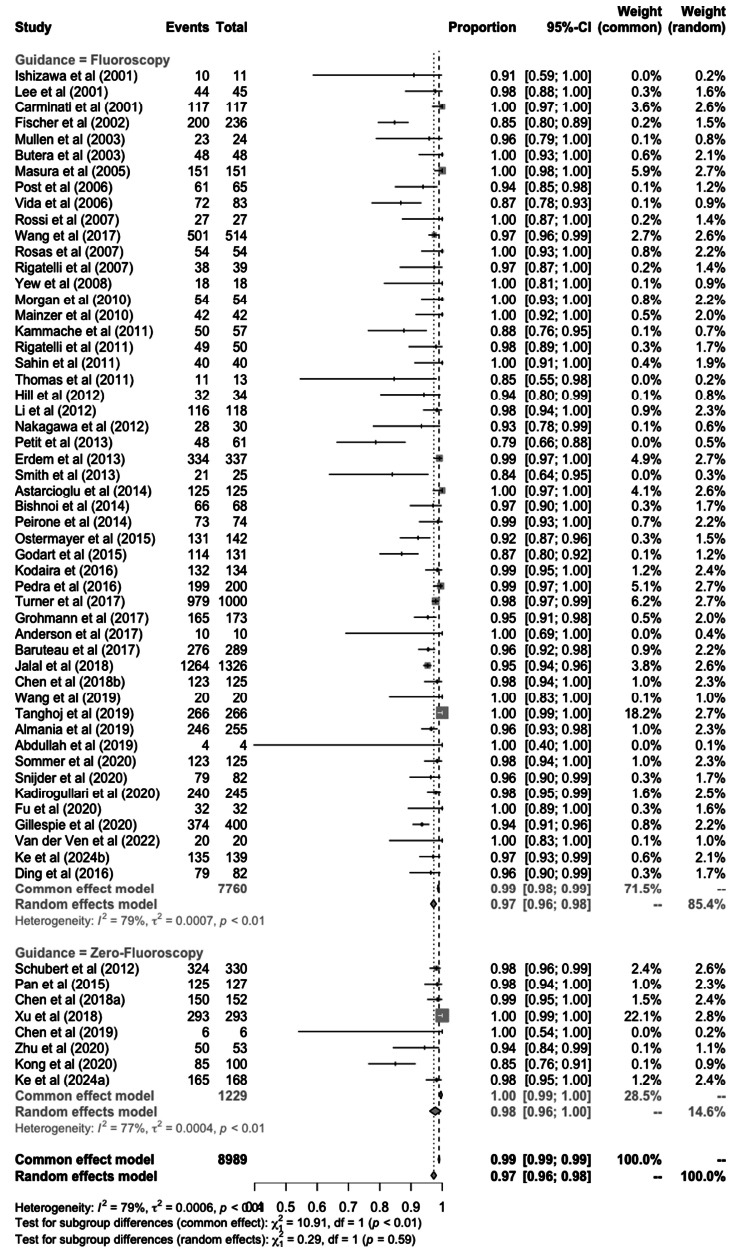
Proportional meta-analysis of percutaneous ASD closure success rate.

**Fig. (6):**
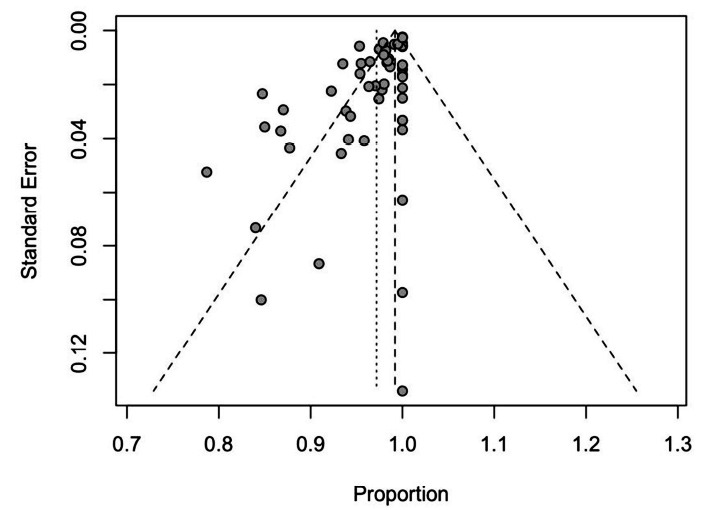
Funnel plot and egger’s regression test on determining publication bias.

**Table 1:** Major complications of percutaneous asd closure.

**Major Complications**	**Proportion (95% CI)**	**Heterogeneity (I^2^)**	**Proportion (95% CI)**	**Heterogeneity (I^2^)**
	**Fluoroscopy**		**Non-Fluoroscopy**	
Device embolization	1% [0-1%]	44%	-	-
AV block	1% [0-1%]	41%	0% [0-2%]	-
Arrhythmia	3% [1-4%]	0%	4% [2-7%]	-
Residual leak	12% [0-28%]	93%	5% [0-10%]	73%

## Data Availability

The authors confirm that the data supporting the findings of this study are available within the article.

## LIST OF ABBREVIATIONS

ASD: Atrial Septal Defects
CHD: Congenital Heart Disease
PRISMA: Preferred Reporting Items for Systematic Reviews and Meta-Analyses
STROBE: Strengthening the Reporting of Observational Studies in Epidemiology

## STANDARDS OF REPORTING

PRISMA methodology and guidelines were followed.

## SUPPLEMENTARY MATERIAL

PRISMA checklist and supplementary material are available on the publisher's website along with the published article.
